# Construction of a New Probe Based on Copper Chaperone Protein for Detecting Cu^2+^ in Cells

**DOI:** 10.3390/molecules29051020

**Published:** 2024-02-27

**Authors:** Jing Ren, Lin Li, Hongfei Han, Yi Chen, Ziying Qin, Zhen Song

**Affiliations:** 1 Laboratory of Protein Based Functional Materials of Shanxi Province, Taiyuan Normal University, Jinzhong 030619, China; 2 Department of Chemistry, Taiyuan Normal University, Jinzhong 030619, China

**Keywords:** probe, protein, copper(II) ion

## Abstract

Biomacromolecular probes have been extensively employed in the detection of metal ions for their prominent biocompatibility, water solubility, high selectivity, and easy modification of fluorescent groups. In this study, a fluorescent probe FP was constructed. The probe FP exhibited high specificity recognition for Cu^2+^. With the combination of Cu^2+^, the probe was subjected to fluorescence quenching. The research suggested that the probe FP carried out the highly sensitive detection of Cu^2+^ with detection limits of 1.7 nM. The fluorescence quenching of fluorescamine was induced by Cu^2+^ perhaps due to the PET (photoinduced electron transfer) mechanism. The FP-Cu^2+^ complex shows weak fluorescence, which is likely due to the PET quenching effect from Cu^2+^ to fluorescamine fluorophore. Moreover, the probe FP can be employed for imaging Cu^2+^ in living cells. The new fluorescent probe developed in this study shows the advantages of good biocompatibility and low cytotoxicity. It can be adopted for the targeted detection of Cu^2+^ in cells, and it has promising applications in the mechanism research and diagnosis of Cu^2+^-associated diseases.

## 1. Introduction

Fluorescence-based optical sensors have been considered a highly sensitive detection method over the past few years. The common fluorescence signal change mechanisms comprise internal charge transfer (ICT) [1], fluorescence resonance energy transfer (FRET) [2], photoinduced electron transfer (PET) [3], aggregation-induced emission effect (AIE) [4], and so on. Of all possible detection mechanisms, PET is one of the most important mechanisms for developing fluorescent probes and biosensors [5]. PET has been frequently employed for constructing numerous fluorescent probes to detect various species that are of considerable biological importance [6,7,8]. A PET probe usually consists of the receptor unit and the fluorophore unit. The receptor unit contains N, O and S atoms used for recognizing the ion. The fluorophore unit is used for translating the host–guest recognition into the fluorescence signal, which usually contains pyrene, cyanine, naphthalimide, and fluorescein [9,10,11,12].

The recognition of metal ions has received attention because they play an important role in biological and environmental processes [13,14]. In the human body, Cu^2+^ is the third abundant metal transition ion that plays crucial roles in numerous important physiological processes [15]. Copper at low concentration levels plays an important role in the functioning of various enzymes. These enzymes are involved in critical processes such as respiration [e.g., cytochrome c oxidase (CcO)], electron transfer/substrate oxidation and iron uptake (ceruloplasmin), pigmentation (tyrosinase), antioxidant defence (Cu/Zn superoxide dismutases), neurotransmitter synthesis and metabolism (dopamine b-hydroxylase, peptidylglycine a-amidating monooxygenase), epigenetic modification (lysyl oxidaselike 2), and handling of dietary amines (copper amine oxidases) [16]. On the other hand, as reported, copper is closely connected with a series of diseases, such as Menkes and Wilson’s diseases, Alzheimer’s, Parkinson’s, prion, and Huntington’s diseases, familial amyotrophic lateral sclerosis and metabolic disorders such as obesity and diabetes [17,18,19]. As per the World Health Organization, the permissible level of Cu^2+^ in drinking water is below 31.5 μM [20]. Consequently, the in situ monitoring of the Cu^2+^ level in environmental or biological samples is important. More recently, copper has also been found to regulate cancers that operate through widely observed oncogenic BRAF mutations and to influence tumour growth [21,22].

Traditional Cu^2+^ detection methods include atomic absorption spectrometry (AAS), atomic emission spectrometry (AES), inductively coupled plasma mass spectrometry (ICP-MS), spectrophotometry and voltammetry [23,24,25]. The above-mentioned detection methods have the advantages of high sensitivity and good selectivity, but they generally have the disadvantages of complicated operation, tedious sample pretreatment, long-time consumption, and high detection cost. The fluorescent analysis strategy, because of the high sensitivity, simple operation, and fast detection speed of fluorescence technique, has attracted great interest. In previous years, multifarious fluorescent materials have been designed and synthesized, such as inorganic fluorescent metal nanoparticles and organic fluorophores [26,27]. Undoubtedly, distinct advances for biochemical sensing have been made by these developed fluorescent methods. However, tedious synthesis, purification, and complicated modification are generally required for the preparation of these fluorescent probes [28,29,30,31]. In particular, most fluorescent materials have low-biocompatibility and even biotoxicity. Thus, it is necessary to construct “green” probes for biochemical sensing based on more biocompatible materials, with environmental friendliness, rapidity, and simplicity [32].

Biomacromolecular probes have been extensively used in the detection of metal ions due to their prominent biocompatibility, water solubility, high selectivity and easy modification of fluorescent groups [33,34,35,36,37]. Copper trafficking metallochaperone is a kind of protein which can transport copper to key enzymatic sites in the cytoplasm. Here, we show that a copper resistance protein from *E. coli*, which was encoded by an *E. coli* resistance plasmid. The plasmid was collected from *E. coli* existing in the gut flora of pigs. Copper sulphate was added into the diet of these pigs as a kind of growth promotant. The pco gene cluster takes charge of encoding the specific copper resistance of such plasmid, which consists of seven genes, namely pcoABCDRSE. PcoC refers to a soluble copper chaperone protein composed of 103 amino acids. The crystal structure of PcoC (PDB1lyq) shows that it is a barrel structure composed of β-sheets [38,39]. The environment in the barrel is quite hydrophobic, and the barrel contains a tyrosine and a tryptophan residue, respectively. Previous research suggested that the N and C terminals of the PcoC have Cu^2+^ and Cu^+^ binding sites, respectively. PcoC binds Cu^2+^ through water and histidines (≥2) in a site with tetragonal distortion. At the other side of the molecular opposite from the Cu^2+^ binding site, the methionine loop was the binding site for Cu^+^. As revealed in the EXAFS studies about the protein regarding the Cu^+^ form, a three-coordinate Cu^+^ existed in an environment in line with the binding of two methionines and one O- or N-donor. PcoC-Cu^2+^ can be reduced to Cu^+^-PcoC by the common reductors such as ascorbic acid and reduced glutathione in organisms [40,41]. PcoC is termed the redox switch of the *E. coli* copper regulation system. The hydrophobic bucket exhibits the property of accommodating small organic molecules. Studying the interaction between small molecules and copper chaperones takes on critical significance in elucidating the regulatory mechanism of copper in organisms.

Fluorescamine refers to a biochemical reagent that is effective for primary amines. The complex formed by the reaction with primary amines is a prominent fluorescent substance [42,43]. The reaction is performed at an ambient temperature, and the medium can be an aqueous solution or organic solution. The reaction conditions are very mild. The most fascinating thing is that the excessive amount of fluorescamine that does not react with the primary amine is rapidly hydrolysed into a non-fluorescent water-soluble substance. The complex formed by the fluorescamine and biological macromolecules (e.g., proteins) possesses fluorescence properties, which provide a basis for the rapid and sensitive determination of amino acids, peptides and proteins [44].

**Scheme 1:**
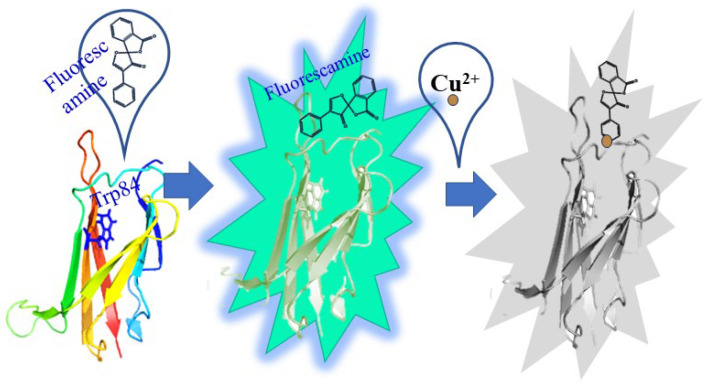
FP-based fluorescent detection of Cu^2+^.

Copper chaperone protein PcoC not only has the advantages of prominent biocompatibility, water solubility and easy modification of fluorescent groups, but also has Cu^2+^ and Cu^+^ binding sites. In addition, it can bind copper ions with high selectivity. Fluorescamine can be combined with biological macromolecules such as proteins to generate fluorescent derivatives. Based on this, we developed a new fluorescent probe likely due to the PET (photoinduced electron transfer) mechanism that can specifically recognize Cu^2+^, and is successfully used for the detection of Cu^2+^ in cells. The detection principle is shown in Scheme 1.

## 2. Results

### 2.1. Construction of the Probe FP

**Figure 1:**
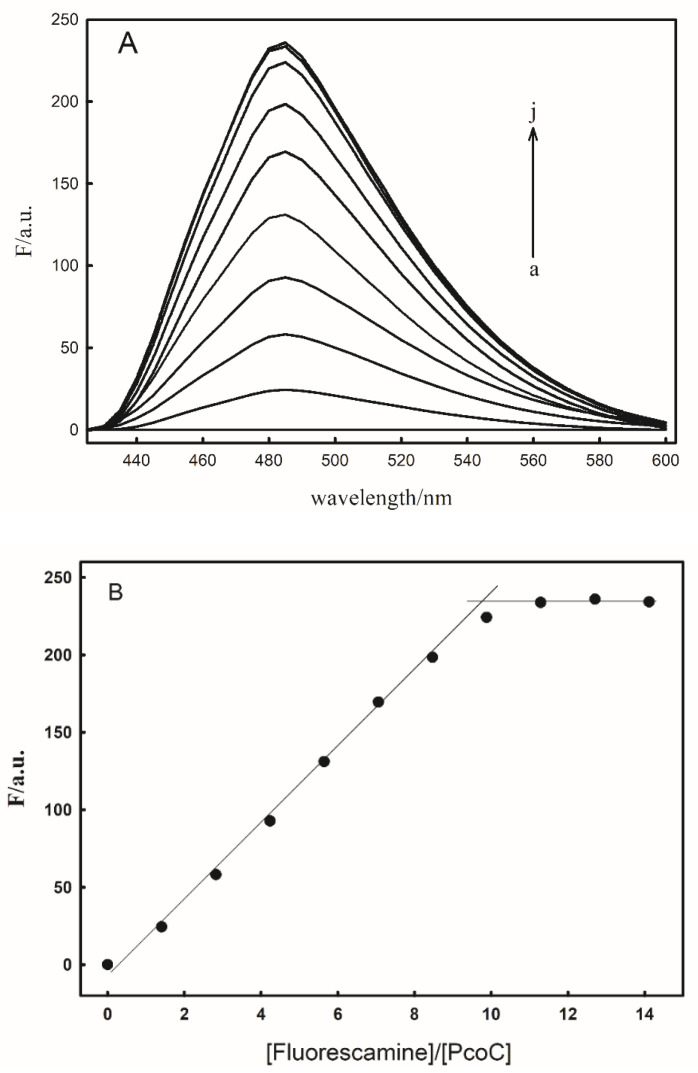
(**A**) The fluorescence spectra of PcoC titrated with fluorescamine; the concentration of fluorescamine from a to j was 0, 0.04, 0.08, 0.12, 0.16. 0.21, 0.25, 0.29, 0.33, and 0.37 mM, respectively. (**B**) The fluorescence intensity monitored at the maximum fluorescence emission wavelength as a function of the [fluorescamine]/[PcoC].

Fluorescamine can be effective for primary amines. The complex formed refers to a strong fluorescent substance. Moreover, excessive fluorescamine are rapidly hydrolyzed into non-fluorescent and water-soluble substances. Thus, fluorescamine was adopted to modify the copper chaperone protein PcoC. With the titration of fluorescamine, a fluorescence peak appeared at 485 nm, as shown in Figure 1A. The fluorescence intensity at 485 nm was plotted with the value of [Fluorescamine]/[PcoC], as shown in Figure 1B. It can be seen that when the concentration of fluorescamine reached nearly 10 times that of the copper chaperone protein, the fluorescence intensity at 485 nm reached its maximum. To explain this phenomenon, we analysed the structure and amino acid sequence of the PcoC. The acid sequence and structure of PcoC was shown in Supporting Information Figure S1. Besides reacting with primary amines at the N-terminus of the PcoC, fluorescamine also reacted with amino acid residues such as lysine, glutamine, asparagine and arginine, which contain primary amine side chains. In the amino acid sequence of PcoC, the total number of amino acids lysine, glutamine, asparagine and arginine reached about 16, as shown in Supporting Information Figure S1. This may be the reason for the binding ratio between fluorescamine and PcoC, which reached 10. From the above findings, it can be seen that the maximum fluorescence emission peak of the fluorescent probe Fluorescamine-PcoC (FP) was at 485 nm.

### 2.2. Optimization of Reaction Conditions

#### 2.2.1. The Best Reaction pH

**Figure 2:**
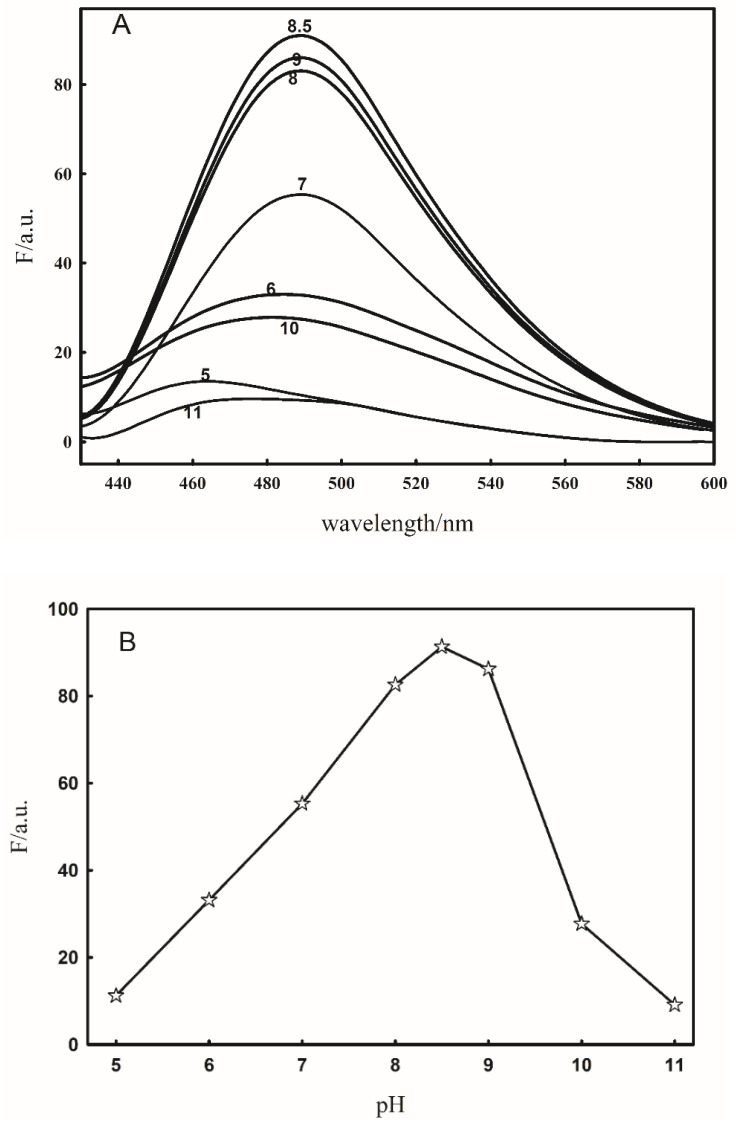
(**A**) The fluorescence spectra of the probe FP with different pH values. (**B**) The effects of pH on the fluorescence intensity of the probe FP.

The fluorescence spectra of the fluorescent probe FP were scanned under different pH conditions, as shown in Figure 2A. Obviously, the fluorescence intensity of the fluorescent probe FP was correlated with the acidity of the solution. When the pH was less than 6 or the pH exceeded 10, the fluorescence intensity was weak. The fluorescence intensity at different pH values was plotted against the pH value, as shown in Figure 2B. It can be seen that the fluorescence intensity reached maximum and relatively stable levels in the range of 7.5 to 9.5, and the fluorescence intensity is the largest at 8.5. The subsequent experiments were performed under pH 8.5.

#### 2.2.2. The Best Reaction Time

To detect the response time of the probe FP to Cu^2+^, the fluorescence emission spectra of the probe were scanned at different times after the addition of copper ions, and the fluorescence intensity was plotted at 485 nm against time, as shown in Supporting Information Figure S2. As depicted in the figure, after Cu^2+^ was added in the probe FP solution, the fluorescence of the probe FP was quenched, and the reaction was stable in 2 min. Thus, the reaction time was selected as 2 min.

### 2.3. Interaction between Fluorescent Probe FP and Cu^2+^

**Figure 3:**
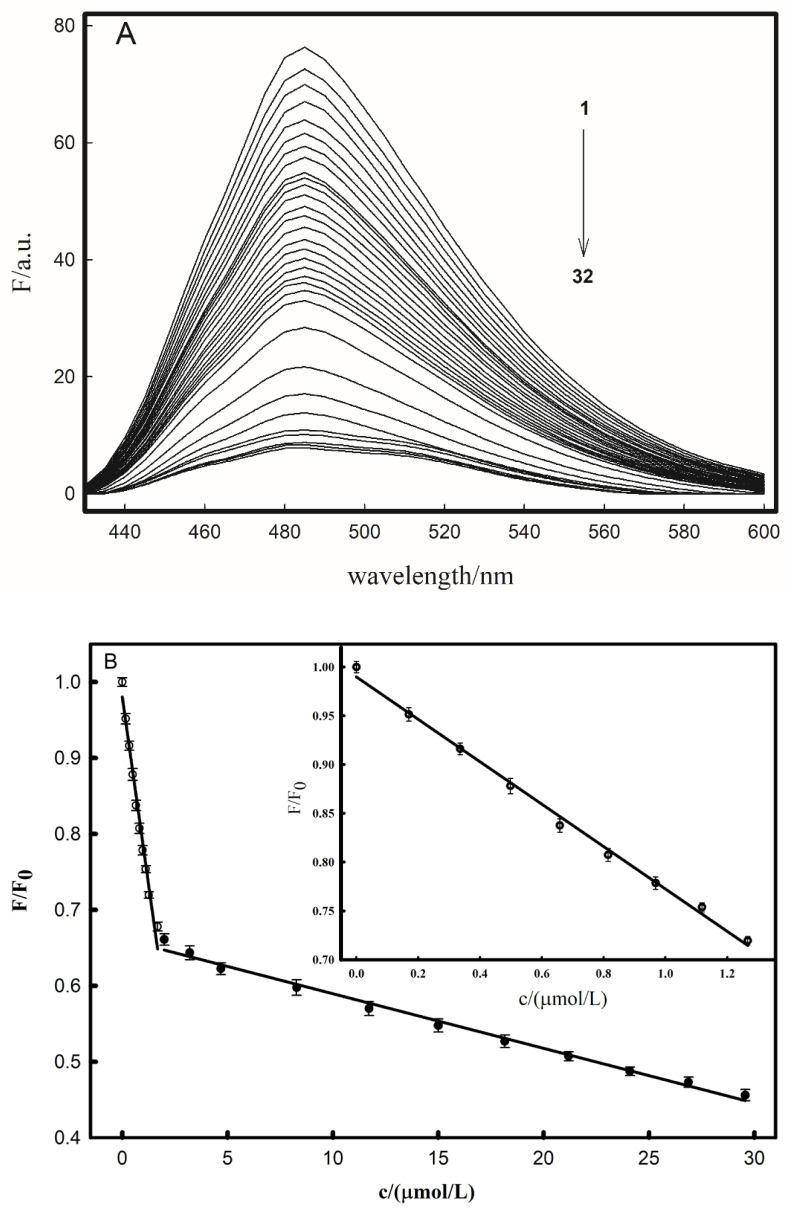
(**A**) The fluorescence quenching spectra were recorded for the probe FP in the presence of various concentrations of Cu^2+^. (**B**) The fluorescence intensity monitored at the maximum fluorescence emission wavelength as a function of the concentrations of Cu^2+^. The concentration of the probe FP was 3.5 × 10^−5^ mol/L.

Under the optimal reaction conditions, Cu^2+^ was titrated to the fluorescent probe FP solution. As depicted in Figure 3A, the fluorescence intensity of the fluorescent probe FP gradually decreases with the increase in the concentration of Cu^2+^. When the concentration of Cu^2+^ reached the millimolar range, the fluorescence intensity of FP no longer declined with the increase in Cu^2+^ concentration. Figure 3B can be obtained using the value of F/F_0_ to plot the concentration of Cu^2+^. As depicted in Figure 3B, there are two good linear relationships between the concentration of copper ion and fluorescence intensity (F/F_0_). The two linear ranges were 0.0002–1.2 μmol/L and 2.0–30 μmol/L, respectively. When the concentration of Cu^2+^ is relatively high, it may cause protein to form an aggregate. These may be the reason why the quenching process is bimodal. The corresponding linear regression equation was expressed as F/F_0_ = 0.99–0.22c, and the linear correlation coefficient was 0.9976. The other equation was F/F_0_ = 0.67–0.0072c; the linear correlation coefficient was 0.9949. In accordance with the three-times signal-to-noise ratio, the detection limit of copper ion was estimated as 1.7 nmol/L. As revealed by the above results, the probe FP can efficiently detect Cu^2+^.

Because PcoC has binding sites of both Cu^+^ and Cu^2+^, and fluorescamine binds to PcoC with the binding ratio of ~10, detecting Cu^+^ can be possible. The fluorescence spectra of the FP-Cu^2+^ complex under different concentrations of GSH were detected. In addition, the corresponding figure and results were added in Supporting Information Figure S3. As shown in Supporting Information Figure S3, with the increase in the concentration of GSH, the fluorescence intensity of the FP-Cu^2+^ complex increased gradually, and the reaction was stable within 10 min.

### 2.4. Selectivity of Fluorescent Probe for Cu^2+^

**Figure 4:**
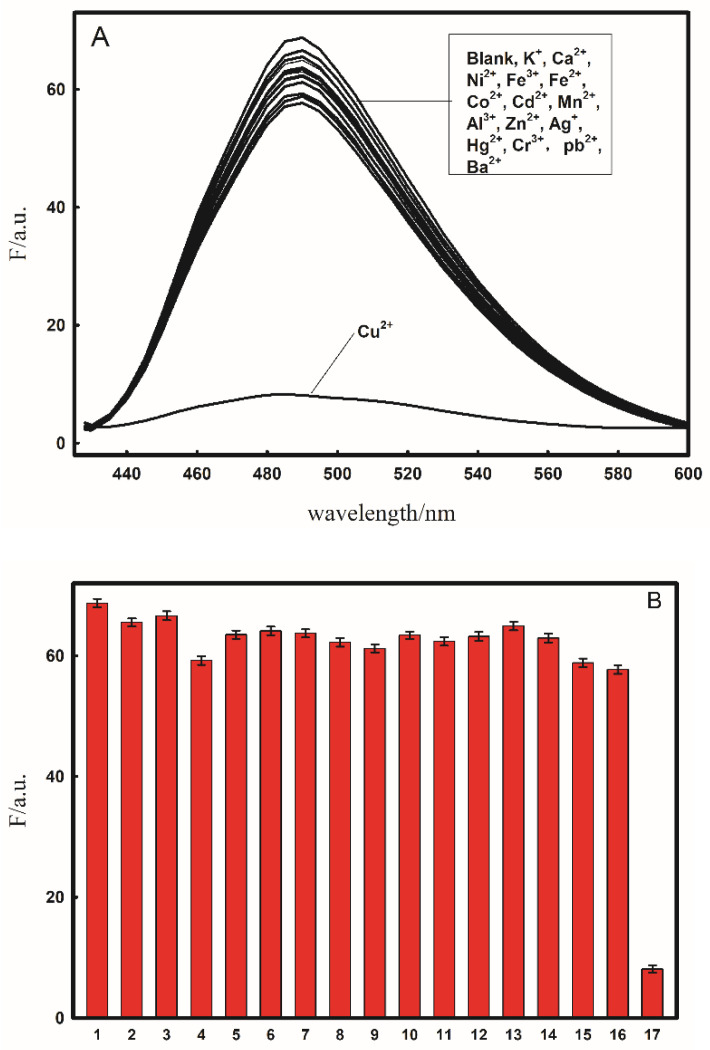

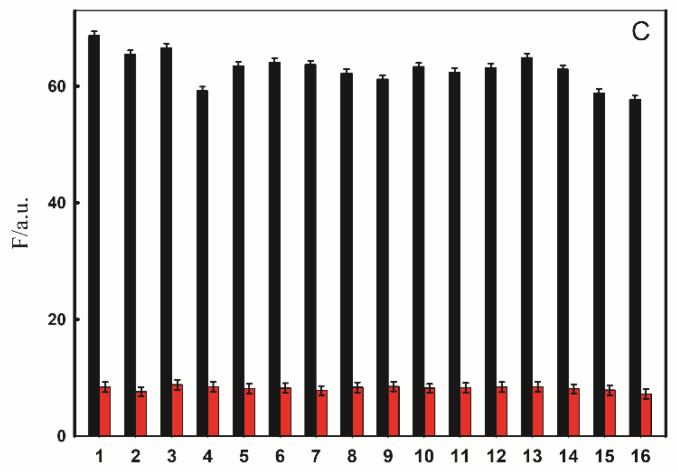
(**A**) The fluorescence spectra of the probe FP with various metal ions. (**B**) The fluorescence intensity of the probe FP in the presence of various metal ions. (**C**) The fluorescence intensity of the probe FP in the presence of other metal ions (black) and together with Cu^2+^ (red). The concentration of the probe FP was 3.5 × 10^−5^ mol/L. (**B**): 1. Blank; 2. K^+^, 3. Ca^2+^, 4. Ni^2+^, 5. Fe^3+^, 6. Fe^2+^, 7. Co^2+^, 8. Cd^2+^, 9. Mn^2+^, 10. Al^3+^, 11. Zn^2+^, 12. Ag^+^, 13. Hg^2+^, 14. Cr^3+^, 15. pb^2+^, 16. Ba^2+^; 17. Cu^2+^. (**C**): 1. Blank; 2. K^+^ + Cu^2+^, 3. Ca^2+^ + Cu^2+^, 4. Ni^2+^ + Cu^2+^, 5. Fe^3+^ + Cu^2+^, 6. Fe^2+^ + Cu^2+^, 7. Co^2+^ + Cu^2+^, 8. Cd^2+^ + Cu^2+^, 9. Mn^2+^ + Cu^2+^, 10. Al^3+^ + Cu^2+^, 11. Zn^2+^ + Cu^2+^, 12. Ag^+^ + Cu^2+^, 13. Hg^2+^ + Cu^2+^, 14. Cr^3+^ + Cu^2+^, 15. pb^2+^ + Cu^2+^, 16. Ba^2+^ + Cu^2+^.

In order to assess the selectivity of the FP fluorescent probe to Cu^2+^, the fluorescence response of the FP probe with adding different metal ions was examined, as shown in Figure 4A. The value of F/F_0_ declined notably after adding Cu^2+^, while the change in F/F_0_ arising from the introduction of other metal ions (K^+^, Ca^2+^, Ni^2+^, Fe^3+^, Fe^2+^, Co^2+^, Cd^2+^, Mn^2+^, Al^3+^, Zn^2+^, Ag^+^, Hg^2+^, Cr^3+^, pb^2+^, Ba^2+^) was very tiny. In order to better reflect the selectivity of the probe to Cu^2+^, the histogram was adopted to represent the change in fluorescence intensity caused by metal ions, as shown in Figure 4B. As depicted in the figure, the probe FP exhibited high selectivity for copper ions. In addition, the effects of the coexistence ion on the detection of Cu^2+^ by probe FP was indicated. The fluorescence response of the fluorescent probe FP to Cu^2+^ in the presence of other ions is shown in Figure 4C. As depicted in Figure 4C, the F/F_0_ value after adding copper ions and other different metal ions in the FP probe solution is close to the value of adding copper ions, which again indicates that the fluorescent probe FP has high selectivity for Cu^2+^.

### 2.5. Cytotoxicity Study

**Figure 5:**
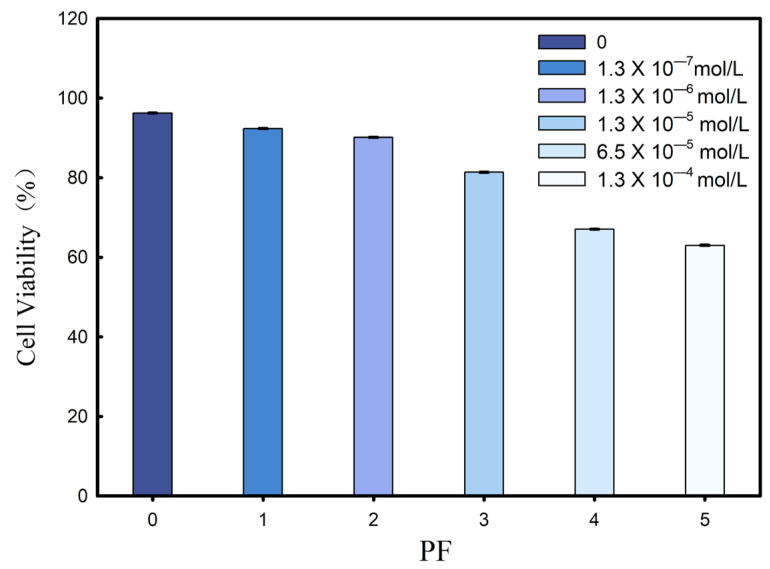
The cytotoxicity of the probe FP with different concentrations.

The cytotoxicity of fluorescent probe FP was studied by the CCK-8 toxicity test. As shown in Figure 5, the micromolar probe concentration has little effect on the cell survival rate, and the cell survival rate is above 90%. When the concentration of the fluorescent probe FP reached 10^−4^ mol/L, the cell survival rate still reached more than 60%. To better determine the cytotoxicity of fluorescent probe FP, we performed fluorescence imaging on cells after 0 h, 7 h and 24 h under different concentrations of probe FP, as shown in Supporting Information Figure S4. It can be seen that with the increase in FP concentration and time, cell morphology became smaller and smaller, and finally became round then gradually died. The above results indicate that the fluorescent probe FP has low cytotoxicity and can be used for cell imaging research.

### 2.6. Cell Imaging Study

**Figure 6:**
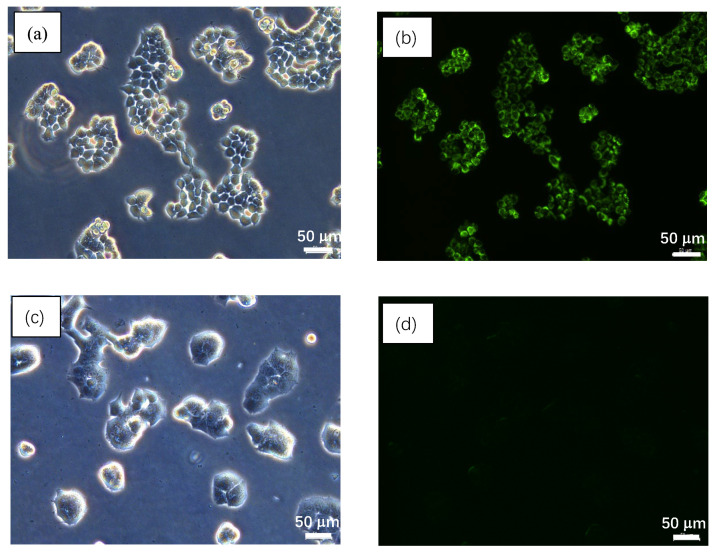
The fluorescence images of MCF-7 cells. Fluorescence images of cells after incubation with FP (**a**), FP and Cu^2+^ (**c**) for 2h. The corresponding bright-field images (**b**,**d**).

To investigate the applicability of the fluorescent probe FP to living cells, Cu^2+^ was detected using the fluorescent probe FP in living cells, as shown in Figure 6. As depicted in Figure 6, when only the fluorescent probe FP existed, MCF-7 cells exhibited strong green fluorescence. MCF-7 cells did not display green fluorescence. The reason why the cells showed green fluorescence perhaps due to the fluorescent probe FP entering the cells. When Cu^2+^ was added to the system, the green fluorescence was significantly quenched. The above-mentioned results fully confirmed that the fluorescent probe FP can be employed for imaging Cu^2+^ in living cells.

## 3. Discussion

PcoC has been reported as a copper chaperone comprising 103 amino acids. It has a hydrophobic barrel structure comprising nine strands of β-sheets, and the center of the hydrophobic barrel includes a tryptophan 84. Under the excitation wavelength of 280 nm, it displayed a fluorescence emission peak at 320 nm. In addition, PcoC can bind to Cu^2+^ with high specificity. Its N-terminus can bind with Cu^2+^, while its C-terminus can bind with Cu^+^. Fluorescamine can be effective for primary amines. The complex with protein has a strong fluorescent substance. On that basis, a fluorescent probe FP was developed, capable of selectively detecting Cu^2+^. Its luminescence mechanism is illustrated in Scheme 1. As depicted in Figure 1A, when the fluorescent amine was introduced to the PcoC solution, a strong emission peak appeared at 485 nm with an excitation wavelength of 390 nm. The reason for this was that the fluorescamine reacted with the primary amine of the protein to form a complex which can produce fluorescence. The fluorescence intensity at 485 nm reached the maximum when the concentration of fluorescamine exceeded 10 times the concentration of PcoC. The possible reason for this was that besides the primary amino group at the N-terminus of the protein, fluorescamine also reacted with amino acid residues with primary amine groups on the surface of the protein. PcoC has many amino acids with primary amine residues. When the Cu^2+^ was added, the fluorescence intensity of the probe at 485 nm tended to decline. As revealed by existing research, Cu^2+^ can bind to the N-terminal of the PcoC, and the binding of Cu^2+^ can reduce the fluorescence intensity of the protein at 320 nm by nearly 60%. Combined with existing studies, the reason for quenching may be due to the PET mechanism. The FP-Cu^2+^ complex shows weak fluorescence, that was likely due to the PET quenching effect from Cu^2+^ to fluorescamine fluorophore.

In brief, a protein-based fluorescent probe FP was developed. Compared with organic small-molecule fluorescent probes, it exhibited prominent properties. The probe can selectively bind to Cu^2+^, resulting in fluorescence quenching of the fluorescent probe FP, thereby achieving high specificity detection of Cu^2+^. In summary, the probe FP developed in this study can recognize Cu^2+^ with high sensitivity and high specificity, likely due to the PET mechanism, and has been successfully used for the detection of Cu^2+^ in living cells, providing a tool for the targeted imaging of Cu^2+^ in cells.

## 4. Experimental Section

### 4.1. Materials and Instruments

Fluorescamine (analytical grade, Shanghai Aladdin Biochemical Technology Co., Ltd., Shanghai, China), dimethyl sulfoxide, sodium tetraborate decahydrate, boric acid (analytical grade, Tianjin Fuyu Fine Chemical Co., Ltd., Tianjin, China), chloride of metal ions (K^+^, Ca^2+^, Mg^2+^, Zn^2+^, Fe^2+^, Mn^2+^, Cu^2+^, Co^2+^, Fe^3+^, Cr^3+^, Hg^2+^, Al^3+^) and nitrate of metal ions (Cd^2+^, Pb^2+^ and Ni^2+^ ions) (analytical grade, Sinopharm Chemical Reagent Co., Ltd., Shenzhen, China) were used.

pH meter (pH SJ-4A), Mettler electronic analytical balance, WGY-10 fluorescence spectrophotometer (Tianjin Gangdong Science and Technology Development Co., Ltd., Tianjin, China), and laser confocal microscope Zeiss LSM 880 were used.

### 4.2. Expression and Purification of PcoC

#### 4.2.1. PcoC Protein Expression

A 427-bp PCR product containing the PcoC gene was synthesized through Sangon Biotech (Shanghai) Co., Ltd., Shanghai, China. The gene fragment covering PcoC was constructed on the vector pET-20b (+) by Xba I and Xho I sites, and the recombinant plasmid pET-20b-PcoC was obtained. The recombinant plasmid pET-20b-PcoC was transformed into *Escherichia coli* BL21 (DE3) for expression. In total, 50 μL of the bacterial solution covering the recombinant plasmid and 5 μL of ampicillin (100 mg/mL) were introduced to 5 mL of the LB culture medium and culture for overnight. The cells were further expanded to 500 mL of the LB culture medium and then shaken in a shaker at 37 °C until the OD reached 0.6~0.8. Then, IPTG was introduced to a final concentration of 0.4 mM, the cells were continued to culture for 3.5 h, centrifuged at 8000 rmp and 4 °C for 15 min for the collection of bacteria.

#### 4.2.2. Purification of the PcoC Protein

The cells were suspended in 20 mM PB buffer at pH 7.0 and then disrupted using an ultrasonic cell disruptor. Afterward, the supernatant was separated and then collected using a high-speed refrigerated centrifuge. The collected supernatant was purified using anion exchange column DE-52 and cation exchange column CM-25 to obtain the target protein PcoC. The purified protein was identified through SDS-PAGE gel electrophoresis. As indicated by the results, the PcoC protein with high purity was generated.

### 4.3. Preparation of Probe Fluorescamine-PcoC (FP)

The fluorescamine was accurately weighed with an analytical balance, dissolved in DMSO and shaken to a constant volume to obtain the fluorescamine solution. The sodium borate buffer solution pH 8.5 was accurately measured in an EP tube with a pipette, and then PcoC (3.5 × 10^−5^ mol/L) and fluorescamine solution (2.47 × 10^−4^ mol/L) were taken with a pipette. The mixed solution was placed at 4 °C for half an hour and then placed at −20 °C for storage. Excessive fluorescamine reagents are hydrolysed into non-fluorescent substances. Therefore, there is no need to deal with excessive fluorescamine. The probe preparation process is not only fast, but also simple to operate.

### 4.4. Fluorescence Spectrum Analysis

The fluorescence emission spectrum of the fluorescent probe FP was obtained using a WGY-10 fluorescence spectrophotometer. A sodium borate buffer solution pH 8.5 was employed in the experimental process. The fluorescence emission spectrum of the probe was scanned under the excitation wavelength of 390 nm, the excitation slit of 10 nm, and the emission slit of 20 nm.

The detection limits of Cu^2+^ were determined in accordance with the detection limit calculation equation LOD = 3 σ/k and the quantitative detection data of fluorescent probes FP in terms of Cu^2+^, where σ denotes the standard deviation under blank conditions, and k represents the slope of the fluorescence quantitative detection curve. For Cu^2+^, the standard deviation (σ) in the detection limit calculation equation is the standard deviation of the fluorescence data measured when only the fluorescent probe FP exists.

### 4.5. Selection of Optimal pH

pH is a key factor affecting the fluorescence of the system, so the fluorescence spectra of the probe at different pH values were studied. The fluorescence intensity of the system was measured at the excitation wavelength of 390 nm. Each pH value was measured three times in parallel and averaged.

### 4.6. Selection of Reactive Time

To determine the optimal reactive time of the system, the probe FP has a very stable fluorescence spectrum at an ambient temperature. Under the optimal pH conditions, Cu^2+^ was added in the solution of FP, the fluorescence intensity of the system was measured immediately by a fluorescence spectrophotometer, and the fluorescence emission spectrum was recorded every 1 min.

### 4.7. Cytotoxicity

The cytotoxicity of fluorescent probe FP was determined using a CCK-8 kit. MCF-7 cells were cultured in a 5% CO_2_ incubator at 37 °C for 24 h. Subsequently, 0.13, 1.3, 13, 65, and 130 μmol/L fluorescent probes FP solution were added to MCF-7 cells for incubation for 24 h. Cell morphology was observed under a microscope and photos were taken to record at 7 h and 24 h, respectively. Finally, CCK-8 was introduced and cultured for 30 min. The absorbance of each microplate was measured by an enzyme-linked immunosorbent assay to calculate the activity of MCF-7 cells.

### 4.8. Cell Imaging

MCF-7 cells were cultured for 24 h and incubated with a 13 μmol/L fluorescent probe FP for 2 h. The cells were washed with phosphate-buffered saline (PBS) three times and imaged by a fluorescence microscope. Subsequently, 10 μmol/L Cu^2+^ was introduced to the medium and cultured for 1 h. After washing with PBS three times, the cells were imaged by fluorescence microscopy [45,46,47].

## Data Availability

The data presented in this study are available in article and Supplementary Materials.

## Supplementary Materials

The following supporting information can be downloaded at: https://www.mdpi.com/article/10.3390/molecules29051020/s1. Figure S1. The amino acid sequence and the primary amine side chains of PcoC. The lysine, glutamine, asparagine and arginine amino acid residue of PcoC was shown as sphere. Figure S2. The reaction time between the probe FP and the Cu^2+^ Figure S3. (A) The fluorescence restoration spectra were recorded for the FP-Cu^2+^ biosensor in the presence of various concentrations of GSH. The red line shown the initial fluorescence spectra of the probe FP without Cu^2+^. (B) The value of F/F0 as a function of the concentrations of GSH. Figure S4. The MCF-7 cell morphology with different concentration of probe FP, under different incubation time 0 h, 7 h and 24 h. The concentration from 1 to 5 was 1.3 × 10^−7^ mol/L, 1.3 × 10^−6^ mol/L, 1.3 × 10^−5^ mol/L, 6.5 × 10^−5^ mol/L, 1.3 × 10^−4^ mol/L, respectively.
