# Supplementary material for: Construction of a New Probe Based on Copper Chaperone Protein for Detecting Cu2+ in Cells
Source: Molecules. 2024 Feb 27;29(5):1020. doi: 10.3390/molecules29051020 (PMC10935257; doi:10.3390/molecules29051020)
Supplement: Supplementary file 1 [file molecules-29-01020-s001.zip › molecules-2863055-supplementary.pdf]

Construction of a New Probe Based on Copper Chaperone Protein for Detecting Cu^{2+} in Cells

Jing Ren [1,2], Lin Li [1,2], Hongfei Han [1,2], Yi Chen [1], Ziying Qin [1] and Zhen Song [1,2,*]

[1] Laboratory of Protein Based Functional Materials of Shanxi Province, Taiyuan Normal University, Jinzhong 030619, China; 2005294064@163.com (J.R.); lilin@tynu.edu.cn (L.L.); hfhan@tynu.edu.cn (H.H.); chenyi@tynu.edu.cn (Y.C.); zyqin@tynu.edu.cn (Z.Q.)

[2] Department of Chemistry, Taiyuan Normal University, Jinzhong 030619, China

* Correspondence: songzhen@tynu.edu.cn

The caption of figure in supporting information:

Figure S1. The amino acid sequence and the primary amine side chains of PcoC. The lysine, glutamine, asparagine and arginine amino acid residue of PcoC was shown as sphere.

Figure S2. The reaction time between the probe FP and the Cu^{2+}

Figure S3. Fig A The fluorescence restoration spectra were recorded for the FP-Cu^{2+} biosensor in the presence of various concentrations of GSH. The red line shown the initial fluorescence spectra of the probe FP without Cu^{2+}.

Fig B The value of F/F0 as a function of the concentrations of GSH.

Figure S4. The MCF-7 cell morphology with different concentration of probe FP, under different incubation time 0 h、7 h and 24 h. The concentration from 1 to 5 was 1.3×10^{-7} mol/L, 1.3×10^{-6} mol/L, 1.3×10^{-5} mol/L, 6.5×10^{-5} mol/L, 1.3×10^{-4} mol/L, respectively.

Figure S1. The amino acid sequence and the primary amine side chains of PcoC. The lysine, glutamine, asparagine and arginine amino acid residue of PcoC was shown as sphere.

H P E L K S S V P Q A D S A V A A P E K I Q L N F S E N L T V K F S G A K L T M T G M K G M S S H S P M P V A A K V A P G A D P K S M V I I P R E P L P A G T Y R V D W R A V S S D T H P I T G N Y T F T V K

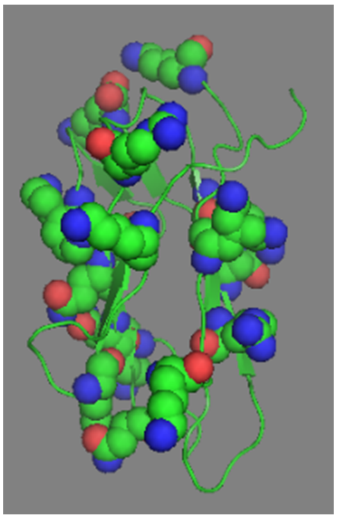

Figure S2. The reaction time between the probe FP and the Cu^{2+}.

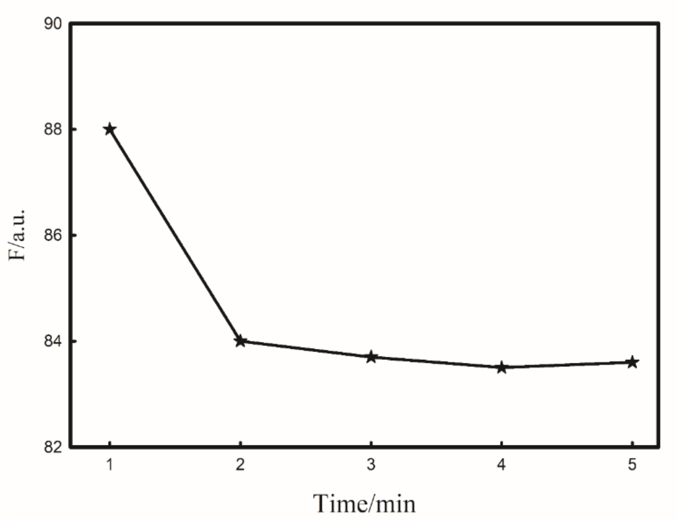

Figure S3. The fluorescence spectra of FP-Cu^{2+} complex under different concentrations of GSH

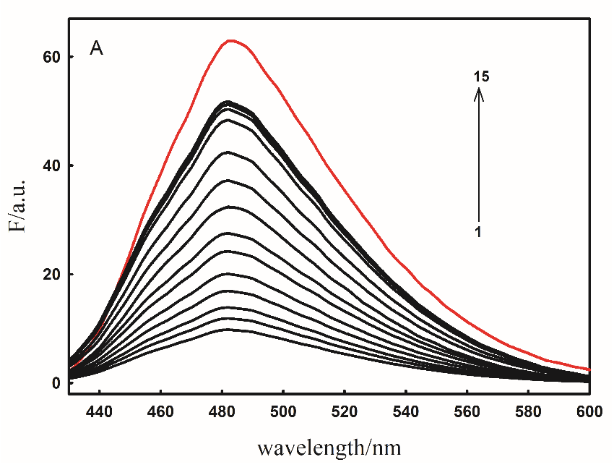

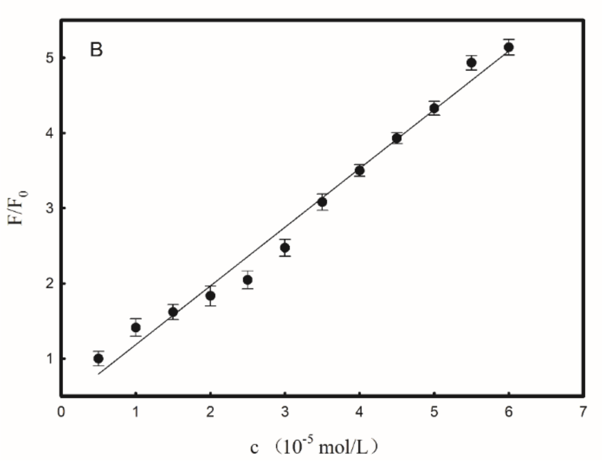

Fig A The fluorescence restoration spectra were recorded for the FP-Cu^{2+} biosensor in the presence of various concentrations of GSH. The red line shown the initial fluorescence spectra of the probe FP without Cu^{2+}.

Fig B The value of F/F_0 as a function of the concentrations of GSH.

Figure S4. The MCF-7 cell morphology with different concentration of probe FP, under different incubation time 0 h、7 h and 24 h. The concentration from 1 to 5 was 1.3×10^{-7} mol/L, 1.3×10^{-6} mol/L, 1.3×10^{-5} mol/L, 6.5×10^{-5} mol/L, 1.3×10^{-4} mol/L, respectively.

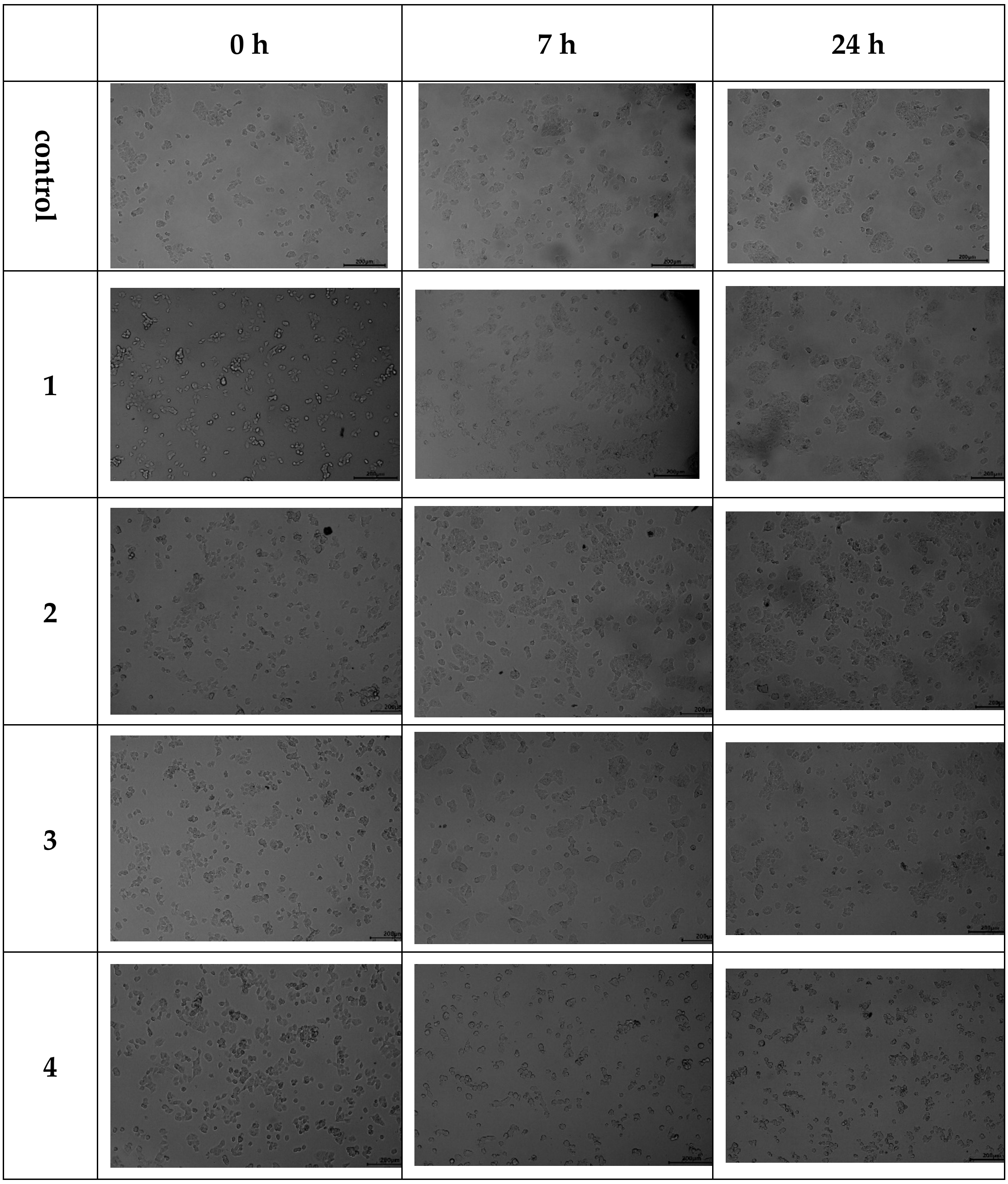

5	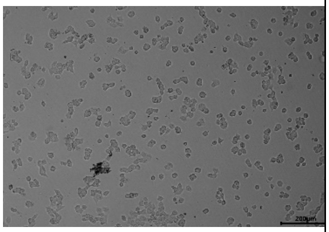	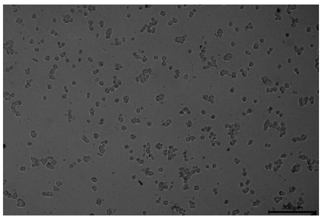	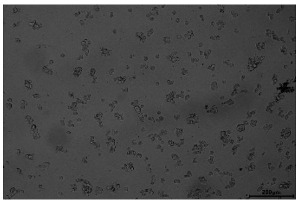
